# Comparative Transcriptome Analyses of *Longissimus thoracis* Between Pig Breeds Differing in Muscle Characteristics

**DOI:** 10.3389/fgene.2020.526309

**Published:** 2020-11-16

**Authors:** Chunbo Cai, Meng Li, Yanwei Zhang, Shan Meng, Yang Yang, Pengfei Gao, Xiaohong Guo, Guoqing Cao, Bugao Li

**Affiliations:** College of Animal Science, Shanxi Agricultural University, Shanxi, China

**Keywords:** differentially expressed gene, genetic type, growth, intramuscular fat, *longissimus thoracis*, pigs

## Abstract

The two breeds, Mashen (MS; a northern China breed) and Large White (LW; a western lean breed) pigs, show important phenotypic differences in growth, adaptability, intramuscular fat (IMF) content, and energy metabolism since early developmental stage. The main aim of this study was the evaluation of *longissimus thoracis* muscle transcriptome profile of both genetic types to identify genes, pathways responsible for their differentiated phenotype. *Longissimus thoracis* of MS and LW pigs were sampled at 0 day (early stage), 90 days (middle stage), and 180 days (later stage) after birth. A total of 3,487 differentially expressed genes (DEGs) were identified at the three time points. Sample clustering analysis revealed the slower growth rate of MS than LW pigs. Gene expression pattern analysis revealed that multicellular organism growth genes (*GHSR*, *EZR*, *FOXS1*, *DRD2*, *SH3PXD2B*, *CSF1*, and *TSHR*) were involved in the fast growth rate of LW pigs at early stage. Furthermore, DEGs (*ACACA*, *ACSF3*, *OXSM*, *CBR4*, and *HSD17B8*) functionally related to fatty acid synthesis revealed that IMF accumulation occurred around 90 and up to 180 days. These DEGs provided valuable resource to study the phenotypic difference in *longissimus thoracis* muscle between MS and LW pigs.

## Introduction

The pig (*Sus scrofa*) served as an important animal for agricultural production, which can provide meat for human consumption. Western pigs have experienced intensive selection, which lead to fast growth rate. However, the meat quality of western pigs is deteriorative due to long-term selection. As a typical lean-type European breed, the Large White (LW) pig is now widely used for commercial production because of its high growth rate and lean meat percentage. In China, there are more pig breeds than in any other country all over the world (Megens et al., 2008). Compared with western pig breeds, Chinese indigenous pig breeds have higher intramuscular fat (IMF), increased tenderness, and better meat quality (Wu et al., 2013; Shen et al., 2014; Liu et al., 2015). Because of the deterioration of meat quality in western pigs, there has been an increasing interest in improving meat quality though use of Chinese indigenous pig breeds. Unfortunately, breed-specific differences in genetics between western pig breeds and Chinese indigenous pig breeds are not fully understood. Therefore, elucidating the genetic differences and biodiversity between western and Chinese pig breeds is important for pig breeding.

High-throughput sequencing technology provides a powerful approach for the analyses of genetic differences and biodiversity in pig breeds. High-throughput sequencing technology mainly includes microarray, genome sequencing, and transcriptome sequencing technology. Many important genes about fatty acid oxidation and biosynthesis have been found in Duroc pigs by combining phenotypic and microarray expression data (Cánovas et al., 2012; González-Prendes et al., 2017, 2019; Cardoso et al., 2018). By genomic analysis among different Chinese and western pig breeds, many growth and meat quality genes were identified (Zhang et al., 2018). Comparative transcriptome analysis between pig breeds plays an important role in studying gene response during tissue development (Freeman et al., 2012). Transcriptome analysis of some extreme phenotypes, such as growth gate and IMF, facilitates to study the gene function and mechanism related to tissue development (Cánovas et al., 2010; Damon et al., 2012; Hamill et al., 2013; Cesar et al., 2015; Wei et al., 2017; Muñoz et al., 2018; Xu et al., 2018). Candidate genes with known effects on skeletal muscle growth were found between purebred and crossbred Iberian pigs by large-scale transcriptome analysis (Óvilo et al., 2014). Researchers applied gene set enrichment analysis (GSEA), partial correlation coefficient with information theory (PCIT), regulatory impact factor (RIF), and phenotypic impact factor (PIF) to better understand the biosynthesis of IMF with the differentially expressed genes (DEGs) between high IMF and low IMF pigs (Cesar et al., 2015). Many important DEGs related to growth and lipid deposition were identified by transcriptome sequencing technology between western and Chinese pig breeds, which can provide many useful data for studying the genetic differences and biodiversity among different pig breeds (Wang et al., 2015; Chen et al., 2017; Liu et al., 2018; Miao et al., 2018; Wu et al., 2020). These studies directly gain deeply insight into the global gene expression profiles of skeletal muscle development in order to reveal the causal genes and biological pathways that influence the growth and meat quality of pigs.

Mashen (MS) pig is a northern China breed. A previous study analyzed the differential phenotype related to growth rate and meat quality between MS and LW pigs (Guo et al., 2019). MS pigs have lower growth rate and higher meat quality than LW pigs (Guo et al., 2019). IMF is one of indicators for meat quality. The content of IMF is associated with juiciness, flavor, tenderness, and desirability of meat (Verbeke et al., 1999). The IMF content of MS pig is higher than that of LW pig (Zhao et al., 2017). Transcriptome analysis of MS and LW pigs was performed to illuminate the molecular mechanisms and candidate genes at 180 days old, which concluded that MS and LW pigs were mainly differentiated by amino acid metabolism, fatty acid metabolism, and skeletal muscle development (Gao et al., 2019). Nevertheless, little is known about the genomic regulation of skeletal muscle development between MS and LW pigs at early, middle, and later developmental stages.

In this study, MS and LW pigs aged 0, 90, and 180 days were selected as models for transcriptome sequencing of *longissimus thoracis* using RNA sequencing technology to identify DEGs and biological pathways influencing skeletal muscle growth, development, and meat quality. Gene Ontology (GO) terms and clustering analysis techniques were adopted to characterize the expression profiles in the muscles of MS and LW pigs at different developmental stages. The aim of this study was to reveal the molecular mechanism underlying the difference of growth rate and meat quality between MS and LW pigs at the transcriptome level.

## Materials and Methods

### Ethics Statement

All animal procedures were done with the Code of Ethics of the World Medical Association (Declaration of Helsinki) and experimented according to American Physiological and World Medical Association General. The methods were based on the College of Animal Science and Veterinary Medicine, Shanxi Agricultural University (Taigu, P.R. China), and the experimental protocols were approved by it. The number of Ethics Committee agreement is SXAU-EAW-P002003.

### Animal Material

A total of 18 healthy male pigs (nine MS pigs and nine LW pigs) were kept under the same feeding and environmental conditions in the Datong Pig Farm in Shanxi Province, China. The 18 boars were divided into six groups. Groups 1 and 2 have three MS and three LW littermate boars aged 0 day. Groups 3 and 4 have three MS and three LW litter boars aged 90 days. Groups 5 and 6 have three MS and three LW litter boars aged 180 days. The three boars of each group are from the same parents. The six boars from groups 1 and 2 were slaughtered at the age of 0 day. The remaining 12 boars were weaned and castrated at the age of 4 weeks. The six boars from groups 3 and 4 were slaughtered at the age of 90 days. The six boars from groups 5 and 6 were slaughtered at the age of 180 days. The samples of *longissimus thoracis* muscle located at last rib were obtained from the carcasses. The samples were collected, snap-frozen in liquid nitrogen, and stored at −80°C for RNA sequence.

### Total RNA Extraction

Total RNA was extracted with TRIzol reagent (Thermo Fisher Scientific Inc., Carlsbad, CA). The total RNA was purified by an RNeasy Micro Kit (QIAGEN, GmBH, Frankfurt, Germany) and RNase-Free DNase Set (QIAGEN, GmBH). The RNA integrity number was determined by an Agilent 2,100 Bioanalyzer (Agilent Technologies, Santa Clara, CA). All samples showed an RNA integrity number above 7.5.

### Library Construction and Sequencing

Ribonucleic acid (2 μg) was depleted of rRNA (Beckman Coulter Inc., Beverly, MA). The eluted RNA was used for sequencing according to Illumina protocols (Illumina Inc., San Diego, CA). First-strand cDNA was synthesized using SuperScript II Reverse Transcriptase (TaKaRa Bio Inc., Dalian, China), and second-strand cDNA synthesis was performed by DNA polymerase I and ribonuclease (RNase). The fragments were purified with an AMPure XP system (Beckman Coulter Inc.); 2.5 μl of USER Enzyme (New England BioLabs, Inc., Ipswich, MA) was added to size-selected, adaptor-ligated cDNA, and the mixture was incubated at 37°C for 15 min followed by 5 min at 95°C. Subsequently, PCR was done with Phusion High-Fidelity DNA Polymerase (Thermo Fisher Scientific Inc., Waltham, MA), Universal PCR primers (Sangon Biotech, Inc., Shanghai, China), and Index (X) Primer (Sangon Biotech, Inc.). The PCR products were purified (AMPure XP system; Beckman Coulter Inc.), and the library quality was determined with the Agilent 2,100 Bioanalyzer system. Finally, the library was sequenced on an Illumina HiSeq 2,500 platform (Illumina Inc.) to generate 2 × 100 bp paired-end reads.

### Validation of Differentially Expressed Genes by Quantitative Real-Time PCR

To evaluate the reliability of gene expression data generated by RNA-Seq, we performed qRT-PCR for 18 randomly selected genes. The total RNAs used in qRT-PCR are generated from the same RNAs used for RNA-Seq. A total of 15 samples were used for qRT-PCR (3 × replicates for 0-day-old LW; 2 × replicates for 90-day-old LW; 2 × replicates for 180-day-old LW; 3 × replicates for 0-day-old MS; 3 × replicates for 90-day-old MS; and 2 × replicates for 180-day-old MS). The cDNA sequence was obtained based on the National Center for Biotechnology Information published sequences, and primers were designed by Primer3web (version 4.0.0). The total RNA was reverse transcribed to cDNA according to the manufacturer’s instructions of the PrimeScript^TM^ RT reagent Kit (TaKaRa, Dalian, China). The qRT-PCR was done using the Mx3000p Real-Time System (Stratagene, La Jolla, CA, United States), with *GAPDH* gene as the internal reference gene. The comparative Ct method was used to analyze the RT-PCR results. The qRT-PCR was performed with three replicates with SYBR^®^ Premix Ex Taq^TM^ (TaKaRa, Dalian, China) using the following parameters: 95°C for 30 s; 40 cycles of 95°C for 5 s, 58°C for 30 s, and 72°C for 30 s. To exclude between-run variations, all samples were amplified in triplicates, and the mean was used for further analysis. The cycle threshold (Ct) was determined using the default threshold settings of the Mx3000p system, and the relative expression of mRNAs was analyzed using the 2^–ΔΔCT^ method.

### RNA-Seq Data Processing

FastQC was used to assess the quality of the sequencing data^1^. The sequences were trimmed to remove the sequencing adaptor; poly A and T tails with Trim Galore^2^ setting default values (stringency of 6 bp) and paired-end reads were kept when both pairs were longer than 40 bp. Filtered RNA-Seq data were aligned to the *Sus scrofa* (Sscrofa11.1)^3^ by hisat2 software with default parameters (Kim et al., 2015). Then the generated SAM files were converted to BAM files by Samtools software (Li et al., 2009). The R package HT-Seq (v0.11.2)^4^ was applied to calculate the count of read pairs against all annotated genes. DEGs were identified using the R (v3.6.1) Bioconductor package, EdgeR (v3.28.0). Gene counts were normalized using the RLE method as imbed in EdgeR (Robinson et al., 2010). Gene expression level was quantified with fragments per kilobase per million (FPKM). DEGs were identified using a generalized linear model likelihood ratio test and Benjamini–Hochberg corrected *P*-value [false discovery rate (FDR) ≤ 0.05 && abs (log2(MS/LW)) ≥ 1].

### Function Annotation of Genes in Pig Genome

Interproscan software (v5.39-77.0) with default parameters was applied to annotated genes in pig^5^ with the protein sequences as input files. KOBAS website was applied to perform Kyoto Encyclopedia of Genes and Genomes (KEGG) analysis (Xie et al., 2011).

### Principal Component Analysis

The package factoextra in R language was applied to perform principal component analysis (PCA). The input files were the relative gene expression level [log2(MS/LW)].

### Gene Ontology Enrichment Analysis

GO describes the functionally related gene sets. Three GO categories (biological process, molecular function, and cellular component) were analyzed in order to achieve a full coverage of the GO spectrum. The GO enrichment analysis for overrepresentation of specific GO terms was performed with the GOseq R package (Young et al., 2010). Multiple testing-corrected *P*-values were also obtained using the Benjamini and Hochberg algorithm, and only GO terms with Benjamini-corrected *P* ≤ 0.05 were selected.

### Protein–Protein Interaction Network Analysis

STRING database integrated protein–protein interaction network of multiple species^6^. STRING database included 4,935,532 interaction edges. We downloaded the interaction database of *S. scrofa* and aligned DEGs identified at 0-, 90-, and 180-day stages to the database in order to understand the interaction relationship of these DEGs at protein level, and modules with tightly interaction relationship were detected by ClusterOne software with default parameters. For better interpretation of the function of each module, GO enrichment analysis was performed.

### Data Availability

The sequencing data have been published in previous studies and were deposited in the Sequence Read Archive with the accession number SRP068558^7^.

## Results

### Sequencing Results

The *longissimus thoracis* muscle of MS and LW pigs were sampled at 0 day (early stage), 90 days (middle stage), and 180 days (later stage) after birth with three biological replicates for each time point. After quality control analysis, RNA-Seq data from 18 libraries were obtained by next-generation sequencing with a total of 429 gigabase (Gb) data obtained after quality control (Supplementary Table 1). The sequencing depth ranged from 17 to 35 Gb. The mapping efficiency ranged from 83.58 to 94.55% and uniquely mapped efficiency ranged from 63.30 to 76.09% (Supplementary Table 1).

The FPKM ≥ 0.5 were used to establish the total number of expressed genes in muscle transcriptome. Approximately 50% of total annotated genes in the Sscrofofa11.1 genome were expressed in the studied samples (an average of 10,992 genes out of 21,279 annotated genes). Based on these results, it was deduced that the RNA-Seq data were of high quality and suitable for subsequent analyses.

### Cluster Analysis of 18 Skeletal Muscle Libraries

**FIGURE 1:**
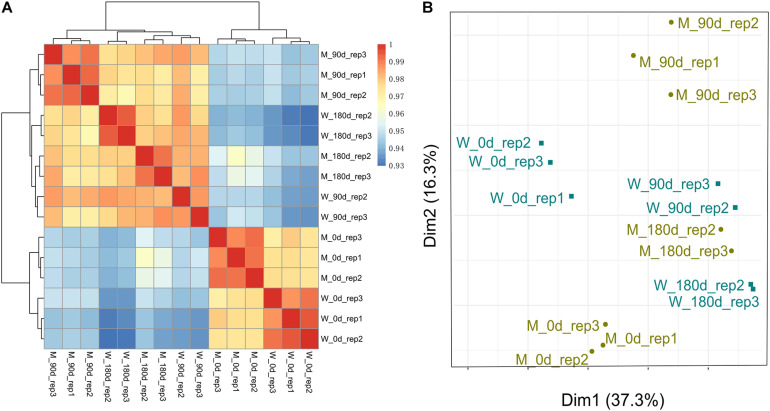
Cluster analysis and PCA of MS and LW pigs. **(A)** Pearson correlation coefficient (PCC) values for each pair of samples and cluster analysis was performed. **(B)** Principal components analysis (PCA). M, Mashen pig; W, Large White pig. 0, 90, and 180 days: pigs aged 0, 90, 180 days, respectively.

To examine the quality of biological replicates, we calculated Pearson correlation coefficient (PCC) values for each pair of samples, and cluster analysis was performed. Three samples (replicate 1 for 90-day-old LW; replicate 1 for 180-day-old MS; and replicate 1 for 180-day-old LW) were clustered far away from their replicated samples (Supplementary Figure 1). Thereafter, the three samples were excluded from our following analysis. Overall, 15 *longissimus thoracis* muscle samples could be divided into two major clusters, which was consistent with muscle development stage (Figure 1A). The first cluster contained breeds sampled at early stage for both MS and LW pigs. The second cluster contained breeds sampled at middle and later development stages. Interestingly, the *longissimus thoracis* muscle samples for LW pigs aged 90 days and MS pigs aged 180 days were clustered together, while other samples were clustered apart (Figure 1A). This result suggested that the slower growth rate of MS pigs compared with LW pigs was confirmed by large-scale transcriptome analysis.

These *longissimus thoracis* muscle samples were also applied for PCA (Figure 1B). The first and second principal components (PC1 and PC2) explain 37.3 and 16.3% variances, respectively. Samples in early, middle, and later development stages could be divided by PC1 (Figure 1B). This result was consistent with Figure 1A.

### Identifying Differentially Expressed Genes During Muscle Development Between Mashen and Large White Pigs

**FIGURE 2:**
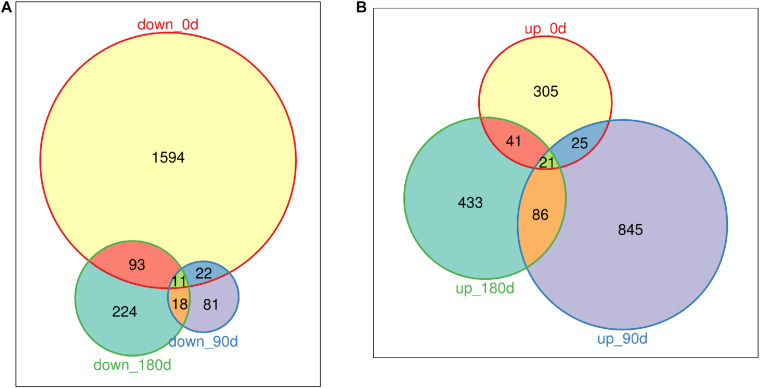
Venn plot of down-regulated genes and up-regulated genes (MS to LW pigs) identified at 0, 90, and 180 days. **(A)** Down-regulated genes. **(B)** Up-regulated genes. M, Mashen pig; W, Large White pig.

DEGs were identified by comparing gene expression data of MS with LW pigs at three time points. In total, 3,487 genes were differentially expressed between the two breeds across the three time points (Figures 2A,B and Supplementary Table 2); 1,720, 132, and 346 genes were down-regulated in MS pigs compared with LW pigs in the three time points, respectively (Figure 2A); 392, 977, and 581 genes were up-regulated (Figure 2B). More genes were down-regulated at 0 day, and more genes were up-regulated at 90 and 180 days (Figures 2A,B). Furthermore, the common and uniquely regulated genes in the three time points were detected. Eleven and twenty one genes were down-regulated and up-regulated in the three time points (Figures 2A,B); 1,594 (92.6%), 81 (61.3%), and 224 (64.7%) down-regulated genes were uniquely identified in the three time point (Figure 2A); 305 (77.8%), 845 (86.5%), and 433 (74.5%) up-regulated genes were uniquely identified in the three time points (Figure 2B). These DEGs served as candidate genes to study the phenotype difference between MS and LW pigs.

### Gene Ontology Enrichment for Differentially Expressed Genes Identified Between the Two Breeds

**FIGURE 3:**
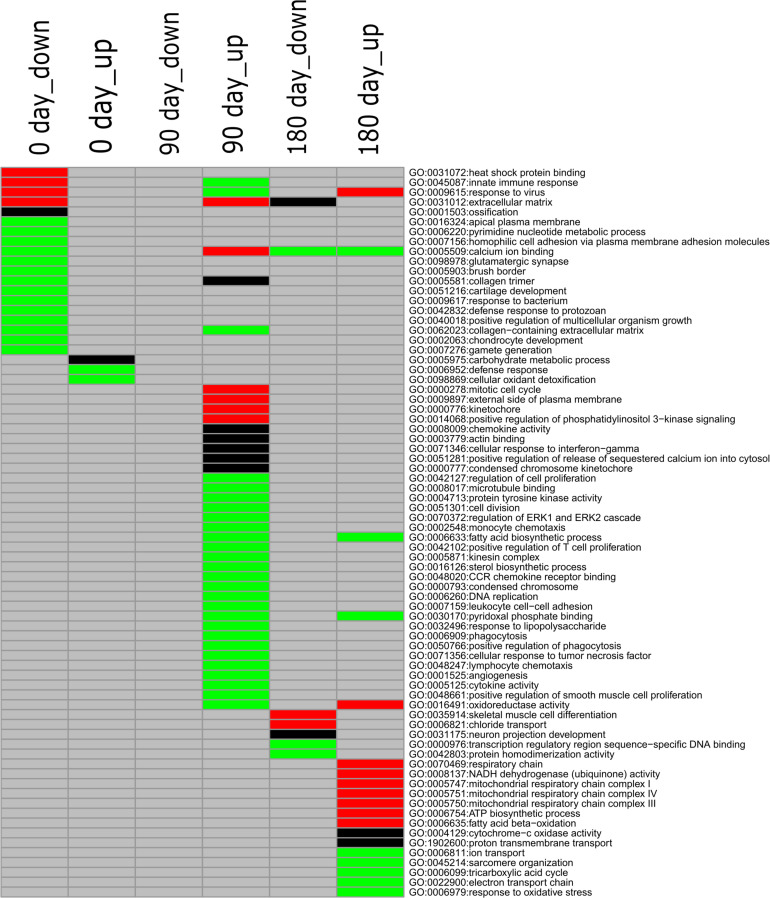
GO enrichment analysis for down-regulated and up-regulated genes (MS to LW pigs) identified at 0, 90, and 180 days. Different colors represent different significance levels: green, FDR < 0.05; black, FDR < 0.01; red, FDR < 0.001; and gray, FDR > 0.05 (i.e., not significant). GO, Gene Ontology; M, Mashen pig; W, Large White pig; FDR, false discovery rate.

To gain deeply insight into the mechanism involved in differentiated development of *longissimus thoracis* muscle between MS and LW pigs, GO enrichment analysis was performed with the up-regulated and down-regulated genes detected at the three time points, and different colored *q*-values were used to visualize varying significance levels (Figure 3 and Supplementary Table 3). The commonly and uniquely overrepresented GO terms enriched at the three analyzed time points were summarized in Figure 3. In total, 73 enriched GO terms were identified in the three time points; 15, 31, and 19 GO terms were uniquely identified in the three time points, while common GO terms were less abundant (Figure 3).

GO:0040018, positive regulation of multicellular organism growth, was enriched in the down-regulated gene sets identified at the early stage in MS pigs compared with LW pigs (Figure 3). *GHSR* (Growth Hormone Secretagogue Receptor), *EZR* (ezrin-radixin-moesin), *FOXS1* (Forkhead Box S1), *DRD2* (Dopamine Receptor D2), *SH3PXD2B* (SH3 And PX Domains 2B), *CSF1* (Colony Stimulating Factor 1), and *TSHR* (Thyroid Stimulating Hormone Receptor) were enriched in it (Supplementary Table 3). We also observed that two GO terms (GO:0001503, ossification; GO:0002063, chondrocyte development) related to skeletal development were enriched in the early stage (Figure 3). Thirty-one GO terms were uniquely enriched in the up-regulated gene set at the middle stage (Figure 3). Among them, GO:0006633 (fatty acid biosynthetic process) and GO:0016126 (sterol biosynthetic process) related to IMF were identified (Figure 3). At the later developmental stage, GO:0035914 (skeletal muscle cell differentiation), GO:0005747 (mitochondrial respiratory chain complexes I), GO:0006099 (tricarboxylic acid cycle), and GO:0022900 (electron transport chain) were overrepresented. These results suggested that differentiated diverse biological pathways were involved in skeletal muscle development in MS and LW pigs.

### Cluster Analysis of Differentially Expressed Genes During Muscle Development

**FIGURE 4:**
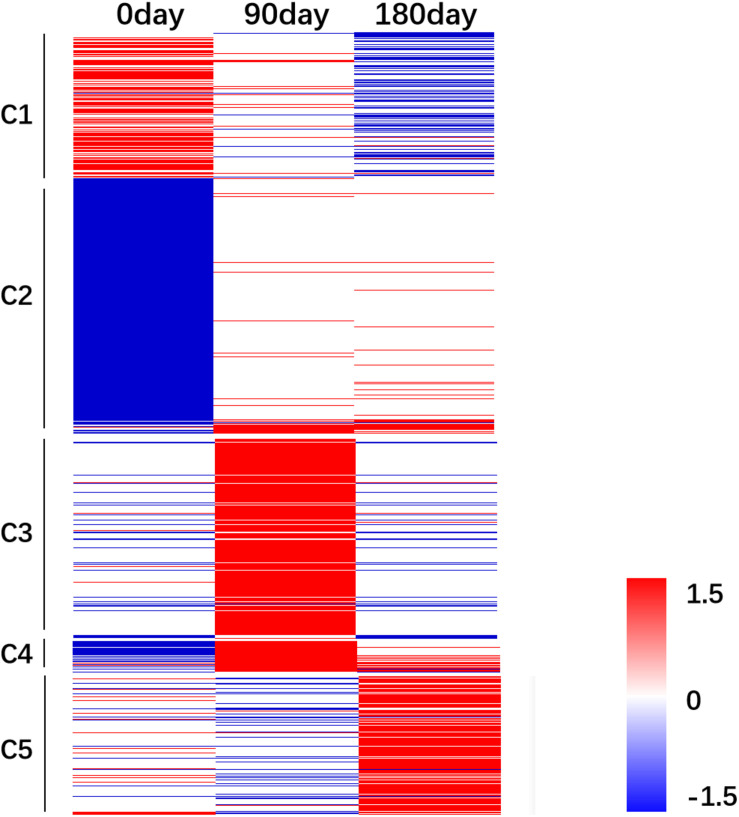
Gene expression pattern analysis. The relative gene expression value [log2(M/W)] was used as input file to perform cluster analysis. Red color indicates genes were up-regulated in MS pig. Blue color indicates genes were down-regulated in MS pig. M, Mashen pig; W, Large White pig.

The relative FPKM values (MS/LW) derived from the RNA-Seq data were used to analyze the gene expression patterns among the three time points. A heat map was generated based on all 3,487 DEGs (Figure 4). To reflect the major trend of gene expression pattern, five clusters were obtained (Figure 4 and Supplementary Table 4). Cluster 1 contained 547 genes, which showed an up-regulated pattern at 0 day, no significant differences at 90 days, and a down-regulated pattern at 180 days. Cluster 2 contained 1,551 genes, which exhibited a down-regulated pattern at 0 day and no significant differences at 90 and 180 days. Cluster 3 contained 758 genes, which showed an up-regulated pattern at 90 days. Cluster 4 contained 109 genes, which showed down-regulated and up-regulated patterns at 0 and 90 days, respectively. Cluster 5 contained 522 genes, which showed an up-regulated pattern at 180 days. These results suggested that *longissimus thoracis* muscle development between MS and LW pigs had a variable effect on gene expression.

### Protein Network Analysis

A protein–protein interaction network analysis was performed for DEGs in order to identify candidate genes and biological pathways potentially affecting the differentiated phenotype of MS and LW pigs. First, a protein–protein interaction network was gathered from the STRING database. Second, DEGs were mapped into the network to get the filtered network, which was further divided into multiple modules (tight interaction gene pairs). In total, 1,322 DEGs (62.6%), 676 DEGs (60.1%), and 595 DEGs (61.2%) identified at 0, 90, and 180 days were mapped to the protein–protein interaction network (Supplementary Table 5), which were further divided into 7, 5, and 11 modules (module size ≥ 10) for each time point based on connection tightness.

GO enrichment analysis was performed for each module in each of the three time points. For module detected at 0 day, the GO terms related to oxidoreductase activity, signal transduction, and extracellular matrix organization were overrepresented in module 1, module 3, and module 7, respectively (Supplementary Table 5). For the subnetwork detected at 90 days, GO:0000922 (spindle pole) and GO:0000278 (mitotic cell cycle) were enriched in module 1 (Supplementary Table 5). The GO terms related to protein dephosphorylation were enriched in module 2. The GO terms related to defense response to virus were enriched in module 4 (Supplementary Table 5). For the subnetwork detected at 180 days, the GO terms related to fatty acid metabolism, steroid hormone receptor, and skeletal muscle cell differentiation were detected in module 2 and module 6 (Supplementary Table 5). The GO terms related to transcription factor activity were also enriched in module 6, which indicated that the activity of muscle cell differentiation was involved in the activity of transcription factors. This protein–protein network enhanced our understanding of *longissimus thoracis* muscle development between MS and LW pigs.

### The Expression Pattern of Genes Related to Fatty Acid Synthesis

**FIGURE 5:**
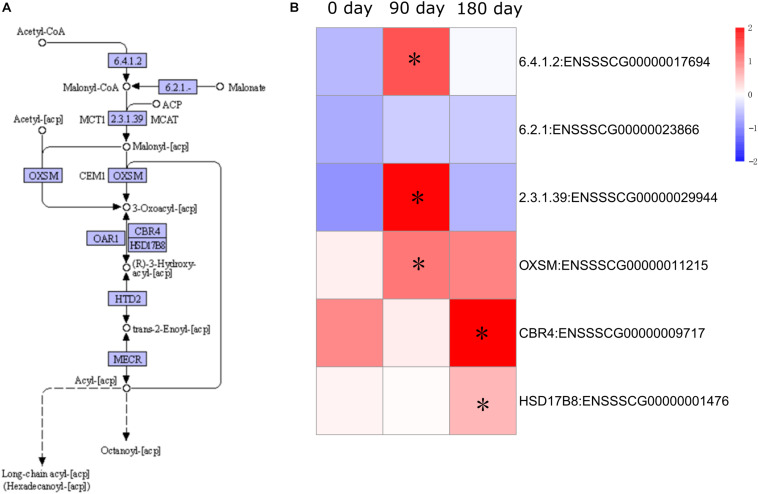
Pathway analysis of fatty acid synthesis. **(A)** KEGG annotation of fatty acid synthesis. **(B)** Gene expression pattern involved in fatty acid synthesis. The asterisk represents the significant level of DEGs (FDR ≤ 0.05). KEGG, Kyoto Encyclopedia of Genes and Genomes; DEGs, differentially expressed genes; FDR, false discovery rate.

Expression of fatty acid metabolism genes has been shown to be an important factor in relation to IMF content (Cagnazzo et al., 2006). To examine the dynamic accumulation of IMF in MS and LW pigs, six genes annotated with fatty acid synthesis by KEGG were collected. Among them, five genes were differentially expressed between MS and LW pigs (Figure 5). Among these five genes, *ACACA* (Acetyl-CoA Carboxylase Alpha), *ACSF3* (Acyl-CoA Synthetase Family Member 3), and *OXSM* (3-Oxoacyl-ACP Synthase, Mitochondrial) were up-regulated at 90 days, and *CBR4* (Carbonyl Reductase 4) and *HSD17B8* (Hydroxysteroid 17-Beta Dehydrogenase 8) were up-regulated at 180 days in MS pig compared with LW pig. *ACACA*, *ACSF3*, and *OXSM* were responsible for the first three steps before 3-Oxoacyl-acp. *CBR4* and *HSD17B8* were responsible for the last two steps after 3-Oxoacyl-acp in the fatty acid synthesis pathway (Figure 5). These results suggested that IMF accumulation occurred around 90 and up to 180 days.

### Validation of RNA-Seq Data by qRT-PCR

**FIGURE 6:**
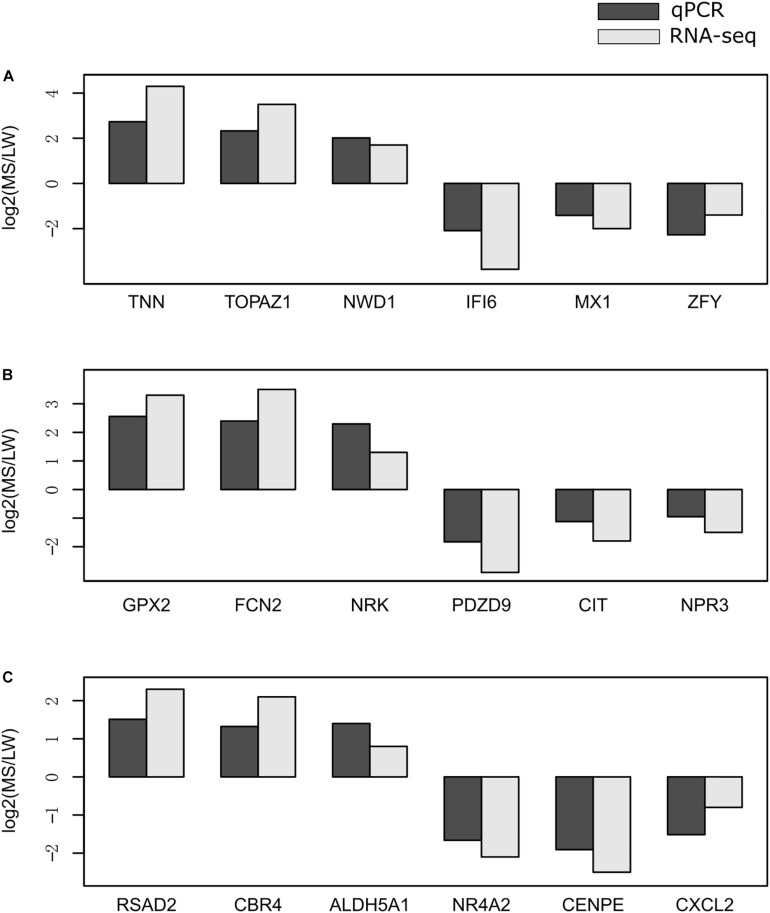
Validation of RNA-Seq data by qRT-PCR. The expression level of randomly selected genes were qvalidated by qRT-PCR with samples collected at 0 day **(A)**, 90 days **(B)**, and 180 days **(C)**. The *X*-axis represents randomly selected genes. The *Y*-axis represents fold change (MS/LW) of the expression level. M, Mashen pig; W, Large White pig.

To validate the reliability of gene expression data generated by RNA-Seq, we randomly selected 18 genes for validation by qRT-PCR. qRT-PCR was performed for the muscle sampled at 0 day (Figure 6A), 90 days (Figure 6B), and 180 days (Figure 6C). *GAPDH* (glyceraldehyde-3-phosphate dehydrogenase) was used as the internal reference gene. Premiers were summarized in Supplementary Table 6. We calculated the fold change value (MS/LW) of gene expression level between MS and LW (Figure 6). Results showed that the trend of gene expression between RNA-Seq and qRT-PCR was similar. The PCC value of gene expression level quantified by RNA-Seq and qRT-PCR was 0.862. This result confirmed that RNA-Seq data analyzed in this study were reliable (Figure 6).

## Discussion

### Effectively Identifying Candidate Genes by Various Omics Data

High-throughput sequencing technology including microarray, genome sequencing, and transcriptome sequencing is widely applied to study the mechanism of growth and meat quality in pigs. *ITGA5* (Integrin Subunit Alpha 5), *LITAF* (Lipopolysaccharide Induced TNF Factor), *TIMP1* (TIMP Metallopeptidase Inhibitor 1), and *ANXA2* (Annexin A2) were identified to participate in lipid synthesis by microarray expression data of *gluteus medius* muscle in high and low backfat thickness Duroc pigs (Cardoso et al., 2018). *FABP3* (Fatty Acid Binding Protein 3), *ORMDL1* (ORMDL Sphingolipid Biosynthesis Regulator 1), and *SLC37A1* (Solute Carrier Family 37 Member 1) were the key roles in carbohydrate and lipid metabolism by microarray expression data of *longissimus thoracis* and *gluteus medius* muscle in Duroc pigs (González-Prendes et al., 2019). By genomic analysis among different Chinese and western pig breeds, *IGF1R* (Insulin Like Growth Factor 1 Receptor) and *SNORA50* (Small Nucleolar RNA, H/ACA Box 50A) were related to growth and meat quality, respectively (Zhang et al., 2018). In this study, we applied RNA-Seq to profile the dynamic gene expression pattern between MS and LW pigs to gain insight into the differentiated mechanism related to skeletal muscle growth and development. Many important genes related to growth rate and lipid biosynthesis were identified. Therefore, microarray, genome sequencing, and transcriptome sequencing are all very important methods for studying the growth rate and meat quality of pigs.

### Intramuscular Fat Accumulation Occurred Around 90 and up to 280 Days

The transcriptome data related to *longissimus thoracis* muscle development were studied in previous research by RNA-Seq, which identified 178 DEGs and several enriched GO terms, especially fatty acid biosynthesis between Laiwu pig, a Chinese indigenous pig breed, and Yorkshire pig at the age of 280 days (Chen et al., 2017). Among these 178 DEGs, 12 (6.74%), 33 (18.54%), and 101 (56.74%) genes were overlapped with DEGs identified in our study at 0, 90, and 180 days, respectively. This result suggested that DEGs identified in muscle at latter stage were similar in different genetic background pigs. Interestingly, GO:0006633 related to fatty acid biosynthesis, which was an important indicator for IMF accumulation (Cagnazzo et al., 2006), was enriched at 90 and 180 days in our study and 280 days in Chen’s work (Chen et al., 2017), which suggested that the differentiated phenotype of IMF accumulation in MS and LW pigs might have occurred at 90 and up to 280 days.

### Candidate Genes Regulating the Intramuscular Fat Content in Pigs

It has been reported that MS pigs showed more abundance of IMF content than LW pigs (Zhao et al., 2017). In this study, many DEGs were likely related to the phenotypes of fat deposition. *ACADM* (acyl-CoA dehydrogenase, C-4 to C-12 straight chain), *UCP3* (uncoupling protein 3), *ACSL1* (acyl-CoA synthetase long-chain family member), and *FADS1* (fatty acid desaturase) enriched in fatty acid metabolism were up-regulated in MS compared with LW pigs at 90 and 180 days (Supplementary Table 2). *ACADM* gene was a functional candidate gene for fatness (Kim et al., 2006). *UCP3* gene was also an important indicator for intramuscular (Nowacka-Woszuk et al., 2008). *ACSL1* gene was significantly associated with polyunsaturated fatty acids in skeletal muscle of beef cattle (Widmann et al., 2011). Overexpression of *ACSL1* could increase fatty acid uptake in 3T3-L1 adipocytes (Zhan et al., 2012). These results indicated that *ACSL1* was a key candidate gene regulating fatty acid metabolism. The expression of *ACSL1* in *longissimus thoracis* muscle of two Chinese native pig breeds (Diannan small ear and Tibet pigs) were significantly higher than that of Yorkshire pigs (Li et al., 2012). Furthermore, RNA-Seq analysis also showed that the expression of *ACSL1* was up-regulated in skeletal muscle of Chinese local Wannanhua pigs and Laiwu pigs compared with Yorkshire pigs (Chen et al., 2017). These results were similar with our results that *ACSL1* was highly expressed in MS pigs compared with LW pigs (Supplementary Table 2). Therefore, the up-regulated expression of *ACSL1* might be associated with higher IMF content in Chinese indigenous breeds compared with western commercial breeds. *FADS1* is an enzyme related to the synthesis of polyunsaturated fatty acids. It has been reported that the polymorphisms of *FADS1* gene was related to fatty acid composition in the brisket adipose tissue of steers (Han et al., 2013), which suggested that *FADS1* gene might play a key role in fatty acid metabolism in skeletal muscle.

### Candidate Genes Responsible for the Slower Growth Rate of Mashen Pigs

A previous study showed that the growth rate of MS pigs was slower than that of LW pigs (Zhao et al., 2017; Guo et al., 2019). We clustered the *longissimus thoracis* muscle tissues sampled at three time points (early, middle, and later development stages). Interestingly, the samples of MS pigs aged 180 days and LW pigs aged 90 days were clustered together (Figure 1A). This result suggested that MS pigs grew slower than LW pigs. Furthermore, GO:0040018 (positive regulation of multicellular organism growth) was enriched at 0 day, and the genes in it were down-regulated in MS compared with LW pigs, which might be functionally related to the slower growth rate of MS pigs (Figure 3). Seven gene (*GHSR*, *EZR*, *FOXS1*, *DRD2*, *SH3PXD2B*, *CSF1*, and *TSHR*) were annotated with GO:0040018. *GHSR* gene controls growth hormone release by inducing a strong stimulatory effect on the endogenous ligand, ghrelin (Zhang et al., 2017). Moreover, the higher expression level of *GHSR* was found in the fast-growing Yorkshire pigs than in the slow-growing Tibetan and Diannan small-eared pigs (Zhang et al., 2017). Therefore, sample clustering analysis and down-regulation of *GHSR* in MS pigs together supported the slower growth rate of MS pigs than LW pigs.

### Expression of Myosin Heavy Chain Family Members Between Mashen and Large White Pigs

Skeletal muscle consists of four myofiber types (types I, IIA, IIX, and IIB), which are characterized by the expression of myosin heavy chain (*MYH*) gene isoforms (Schiaffino and Reggiani, 2011). *MYH7*, *MYH2*, *MYH4*, and *MYH3* are the specific genes of type I, type IIA, type IIB, and type IIX myofibers, respectively (Schiaffino and Reggiani, 2011). In a previous study, the specific gene expression of type I and type IIA myofiber in MS pigs was significantly higher than that in LW pigs, whereas the specific gene expression of type IIB myofiber was higher in LW pig (Guo et al., 2019). In the present study, the expression of *MYH7* in MS pigs was higher than that in LW pigs at 0 day, but there were no significant differences at 90 and 180 days. The content of type I myofiber decreased with age, which might be the cause of no significant difference between MS and LW pigs at 90 and 180 days. Furthermore, there were no significant differences in the expression of *MYH2* and *MYH4* between MS and LW pigs at the three time points (early, middle, and later development stages) in this study. The gene ID of *MYH2* (ENSSSCG00000029441) is the same as *MYH4* annotated in reference Sscrofa11.1, which might lead to no difference with *MYH2* and *MYH4* expression between MS and LW pigs. Interestingly, the expression of *MYH3* was significantly higher in MS pigs than that in LW pigs at the three time points. *MYH3* might play an important role in regulating the switch of myofibers in pigs.

## Conclusion

In this study, RNA-Seq technique was employed to explore the transcriptome difference of *longissimus thoracis* muscle in MS and LW pigs. A total of 3,487 DEGs were identified, which served as an important resource to discover the mechanism and genes involved in skeletal muscle growth rate, meat quality, and energy metabolism. MS pigs grew slower than LW pigs, which supported the sample cluster results and DEGs annotated with multicellular organism growth genes. The expression of fatty acid synthesis genes was higher in MS pigs than that in LW pigs at 90 and 180 days, which might explain the phenotype that more IMF was accumulated in MS pigs than that in LW pigs. In summary, this study gave new sight into the phenotype difference of skeletal muscle in MS and LW pigs, which can provide basic data for the research of Chinese and foreign pig breeds.

## Data Availability Statement

The datasets generated for this study can be found in the Sequence Read Archive with the accession number SRP068558 (https://trace.ncbi.nlm.nih.gov/Traces/sra_sub/sub.cgi?login=pda).

## Ethics Statement

The animal study was reviewed and approved by the Charter of the Animal Ethics Committee of Shanxi Agricultural University.

## Author Contributions

ML, YZ, and SM took the longissimus dorsi of Mashen and Large White pigs. CC, YY, and PG performed data analysis. XG, GC, and BL designed this study. CC, GC, and BL wrote the manuscript. All authors contributed to the article and approved the submitted version.

## Conflict of Interest

The authors declare that the research was conducted in the absence of any commercial or financial relationships that could be construed as a potential conflict of interest.

## Footnotes

1 http://www.bioinformatics.babraham.ac.uk/projects/fastqc/

2 http://www.bioinformatics.babraham.ac.uk/projects/trim_galore/

3 ftp://ftp.ensembl.org/pub/release-98/fasta/sus_scrofa/dna/Sus_scrofa.Sscrofa11.1.dna.toplevel.fa.gz

4 http://pypi.python.org/pypi/HTSeq

5 ftp://ftp.ebi.ac.uk/pub/software/unix/iprscan/5/5.39-77.0/interproscan-5.39-77.0-64-bit.tar.gz

6 https://string-db.org/

7 https://trace.ncbi.nlm.nih.gov/Traces/sra_sub/sub.cgi?login=pda

## Supplementary Material

Supplementary Figure 1 Cluster analysis of all muscle samples.

Supplementary Table 1 Summary of sequencing data.

Supplementary Table 2 Information of DEGs.

Supplementary Table 3 GO information for up-regulated and down-regulated genes at three time points, respectively.

Supplementary Table 4 Gene information in each of 5 clusters.

Supplementary Table 5 Subnetwork information of protein-protein interaction network.

Supplementary Table 6 Information of premiers used for qRT-PCR.
