# Supplementary figures and images for: Comparative Transcriptome Analyses of Longissimus thoracis Between Pig Breeds Differing in Muscle Characteristics
Source: Front Genet. 2020 Nov 16;11:526309. doi: 10.3389/fgene.2020.526309 (PMC7717936; doi:10.3389/fgene.2020.526309)

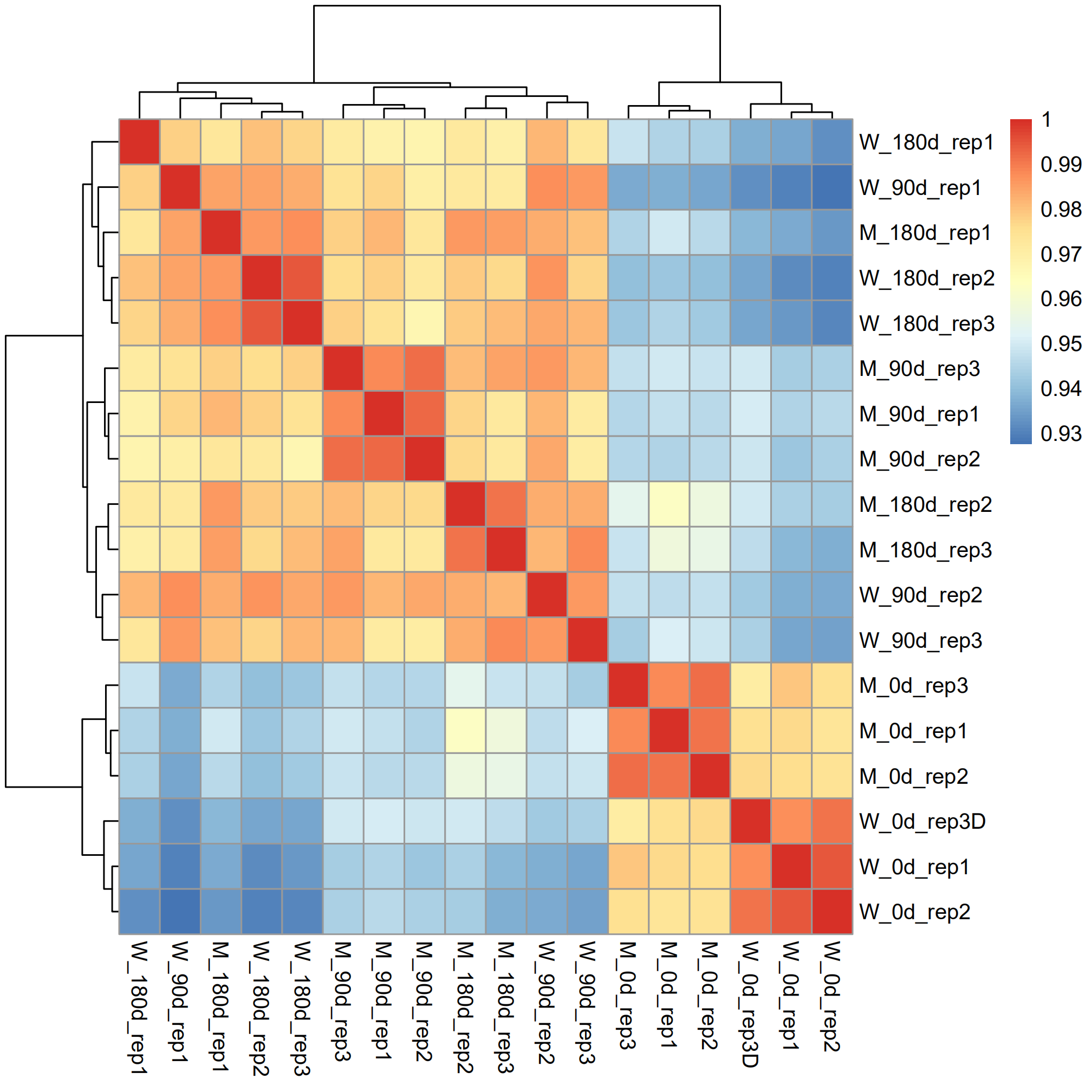

Supplement: Supplementary Figure 1 — Cluster analysis of all muscle samples. [file Image_1.TIF]
