# Supplementary material for: Comparative Transcriptome Analyses of Longissimus thoracis Between Pig Breeds Differing in Muscle Characteristics
Source: Front Genet. 2020 Nov 16;11:526309. doi: 10.3389/fgene.2020.526309 (PMC7717936; doi:10.3389/fgene.2020.526309)
Supplement: Supplementary Table 6 — Information of premiers used for qRT-PCR. [file Table_6.DOCX]

Supplemental table 6 Information of premiers used for qRT-PCR

| Gene Number | Name | primers | Length |
| --- | --- | --- | --- |
| ENSSSCG00000034570 | IFI6 | F: AAGACGCTCTGAGGACAACA  R: TCTGCTTTCGAGTTGCTTGC | 298 Bp |
| ENSSSCG00000012077 | MX1 | F: GTCATCGGGGACCAGAGTTC  R: TCCCGGTAACTGACTTTGCC | 164 Bp |
| ENSSSCG00000012179 | ZFY | F: ACTTACCTTTCAGCGGGCTC  R: AGTAACCCCCTCCGTACCTG | 255 Bp |
| ENSSSCG00000015507 | TNN | F: TGGCAGATATGGGGAGACCA  R: GGGGCGAATCTTCAACTCCA | 100 Bp |
| ENSSSCG00000029268 | TOPAZ1 | F: TGAGCGATCACAGTGCAAGT  R: GCACGCTGTAGCAAACACAA | 114 Bp |
| ENSSSCG00000013865 | NWD1 | F: GAAGCATGGCCTGATGTTCG  R: TGTTTTCCGACACCGGTCAA | 118 Bp |
| ENSSSCG00000002279 | GPX2 | F: CTGGACGGGGAGAAGGTAGA  R: CGGACGTACTTGAGGCTGTT | 224 Bp |
| ENSSSCG00000023333 | FCN2 | F: CAACGACCAGTACGCCTCAA  R: GGCCCGAAACTTCATCTCCG | 190 Bp |
| ENSSSCG00000012546 | NRK | F: CGTAAGAACAGGCTTCGGGT  R: AGTCTCCTTCGGAGTCTGCT | 298 Bp |
| ENSSSCG00000026686 | PDZD9 | F: CTGCAGCAGAGGAACCCAATA  R: ATGGTCTGGGTACGTTTGATTT | 287 Bp |
| ENSSSCG00000009839 | CIT | F: CAGGAGCGATTTACCTGGCA  R: GGGCCTCGCTTGTTAGGG | 136 Bp |
| ENSSSCG00000036201 | NPR3 | F: CAGCTCTCCTTGCAAAGCAT  R: CTCTTCTTGCTGGTTTCGCC | 154 Bp |
| ENSSSCG00000008648 | RSAD2 | F: AAAGCTCTGAACCCTGTCCG  R: CTTCCGCCCGTTTCTACAGT | 243 Bp |
| ENSSSCG00000009717 | CBR4 | F: GGGTTCCATGCTGACGTGTA  R: GTGTGAACAAACCCTGGTGC | 222 Bp |
| ENSSSCG00000001090 | ALDH5A1 | F: CTGTGCACGGATCCTCTTGT  R: ACATTGGCGCTGTCAAACAC | 152 Bp |
| ENSSSCG00000015871 | NR4A2 | F: TCTTGCCACCCAACATGAGG  R: GCCGAGTTACAGGCGTTTTC | 256 Bp |
| ENSSSCG00000023296 | CENPE | F: CCTGAGGAGAGCAGAGGAAAC  R: ACCTCCTTATTGTTCTGTTGATCC | 261 Bp |
| ENSSSCG00000008959 | CXCL2 | F: AGTTTGTCTCAACCCCGCAG  R: CATCAGTTGGCACTGCTCTTG | 78 Bp |
| ENSSSCG00000000694 | GAPDH | F: TCGGAGTGAACGGATTTGGC  R: TGACAAGCTTCCCGTTCTCC | 189 Bp |
